# Paediatric Chordomas

**DOI:** 10.1186/s13023-015-0340-8

**Published:** 2015-09-22

**Authors:** Kévin Beccaria, Christian Sainte-Rose, Michel Zerah, Stéphanie Puget

**Affiliations:** Service de neurochirurgie, hôpital Necker-Enfants Malades, Paris, France; Faculté de médecine, université Paris Descartes, Sorbonne Paris Cité, Paris, France

**Keywords:** Paediatric, Chordoma

## Abstract

Paediatric chordomas are rare malignant tumours arising from primitive notochordal remnants with a high rate of recurrence. Only 5 % of them occur in the first two decades such less than 300 paediatric cases have been reported so far in the literature. In children, the average age at diagnosis is 10 years with a male-to-female ratio closed to 1. On the opposite to adults, the majority of paediatric chordomas are intracranial, characteristically centered on the sphenooccipital synchondrosis. Metastatic spread seems to be the prerogative of the under 5-year-old children with more frequent sacro-coccygeal locations and undifferentiated histology. The clinical presentation depends entirely on the tumour location. The most common presenting symptoms are diplopia and signs of raised intracranial pressure. Sacrococcygeal forms may present with an ulcerated subcutaneous mass, radicular pain, bladder and bowel dysfunctions. Diagnosis is suspected on computerised tomography showing the bone destruction and with typically lobulated appearance, hyperintense on T2-weighted magnetic resonance imaging. Today, treatment relies on as complete surgical resection as possible (rarely achieved because of frequent invasiveness of functional structures) followed by adjuvant radiotherapy by proton therapy. The role of chemotherapy has not been proven. Prognosis is better than in adults and depends on the extent of surgical resection, age and histology subgroup. Biological markers are still lacking to improve prognosis by developing targeted therapy.

## Résumé

Les chordomes pédiatriques sont des tumeurs rares développées à partir de résidus de la notochorde primitive et qui présentent un haut taux de récidive locale. Les chordomes des sujets de moins de 20 ans représentent seulement 5 % de l’ensemble de ces tumeurs, justifiant que moins de 300 cas de chordomes pédiatriques aient été publiés dans la littérature à ce jour. Chez l’enfant, l’âge moyen au diagnostic est de 10 ans avec un ratio garçon-fille proche de 1. Contrairement à l’adulte, la majorité des chordomes pédiatriques sont intra-crâniens, typiquement localisés en regard de la synchondrose sphéno-occipitale. La dissémination métastatique semble être l’apanage des enfants de moins de 5 ans, avec des localisations sacro-coccygiennes et une histologie indifférenciée plus fréquentes. La présentation clinique dépend essentiellement de la localisation de la tumeur. Les symptômes les plus fréquemment retrouvés au diagnostic sont la diplopie et les signes d’hypertension intra-crânienne. Les formes sacro-coccygiennes peuvent se présenter sous la forme d’une masse sous-cutanée ulcérée, ou bien par des douleurs radiculaires ou des troubles vésico-sphinctériens. Le diagnostic est suspecté devant une lésion ostéolytique au scanner, classiquement lobulée et en hypersignal T2 en IRM. Aujourd’hui, le traitement repose sur une résection chirurgicale aussi complète que possible (rarement obtenue du fait de l’envahissement fréquent de structures fonctionnelles par ces tumeurs), suivie d’une radiothérapie adjuvante aux protons. Le rôle de la chimiothérapie n’a pas été démontré. Le pronostic est meilleur chez l’enfant que chez l’adulte, et il dépend de la qualité de la résection chirurgicale, de l’âge et du type histologique. A ce jour, aucun marqueur n’a pu être mis en évidence, qui permettrait de développer des thérapies ciblées et améliorer le pronostic de ces tumeurs.

## Introduction

Paediatric chordomas are rare malignant tumours which involve the cranial base and spinal column. They seem to be different from their adult’s counterparts. The aim of this article is to give an update review of clinical and histopathological features, diagnostic criteria, genetic markers, prognosis factors and current treatment. Data discussed in this review were obtained from a Pubmed search with the terms « paediatric », « pediatric » and « chordoma ».

## Review

### Disease name/synonyms

Chordoma ORPHA178

### Definition

Chordomas are rare tumours arising from primitive notochordal remnants [1]. Their anatomopathological classification is the same for adults and children and distinguishes “classic” (“chordoma NOS” Not Otherwise Specified), “chondroid” and “undifferentiated” chordomas.

Luschka first described the existence of small soft transparent “jelly-like” tumours of the *clivus blumenbachii* (*dorsum sellae*) in 1857 [2], and Ribbert introduced the term “chordoma” thirty years later [3]. While one of the first cases of chordoma was described by Klebs in 1864, the first paediatric cases were reported many years later by André-Thomas [4] and Adson [5] in 1923 and 1935.

The diagnosis of a patient with a chordoma is based on clinical, topographical and radiological (multilobulated, hyperintensity in T2 weighted sequences) findings, and is then confirmed by characteristic histological findings. Chordomas are characterized by their aggressive potential and their frequency of recurrence. Management of these tumours is based on as complete as possible primary resection followed by local irradiation, ideally proton beam therapy.

### Epidemiology

Chordomas comprise 0.2 % of primary brain tumours [6, 7] and less than 5 % of primary bone tumours [6, 8, 9]. They occur in less than 1/1,000,000 population [8, 10], with a peak incidence occurring between the fourth and sixth decades [11–13]. Less than 5 % of chordomas present in the first two decades [11, 14–16].

Among children, the average age at diagnosis was around 10 years [15–20], and the youngest case described was in a neonate with a tumour of the clivus [21]. In contrast to adults [6, 12, 22], sacro-coccygeal chordomas in children occur at a younger age than those in the cranium. For all localisations, the male to female ratio is close to 1 [15, 18].

### Clinical description

#### Anatomical localisation

In the literature, childhood chordomas are clearly distinguished from their adult’s counterparts by their anatomical distribution. Adult chordomas are primarily found in the sacro-coccygeal region [13, 15, 23–25], whereas the majority of paediatric chordomas are intracranial (up to 54 %) [15], characteristically centered on the sphenooccipital synchondrosis [11, 15, 17, 26, 27]. Local destruction of the clivus with extradural compression of the neuroaxis is a characteristic feature. Other locations are anecdotic (gluteal region [28], paranasal sinuses [29], ethmoid and maxillary sinuses [30], temporal bone [31]). Cases of intradural lesions [32–34] and chordomas arising in the paravertebral or paraclival regions without associated bony invasion have also rarely been reported [35, 36].

#### Intracranial chordomas

Impairment of cranial nerve function is the principle presenting feature of chordomas of the cranial base in approximately 60 % of cases [19, 23], the sixth nerve being the most frequently involved (55 to 72 % of cases) [16]. Headaches occur in around 40 % of cases [16, 23], due to increased intracranial pressure (ICP) in 28 % of cases [23]. Long tract signs with a pyramidal syndrome may occur in 36 % of cases [23].

Children less than 5 years present frequently with increased ICP (72 %), long tract signs (43 %) and also lower cranial nerves palsy and torticollis probably due to a more frequent inferior extension of lesions at this age [24]. Older children will present with diplopia or isolated headaches (55 % et 42 % respectively) [21, 23, 24].

#### Sacro-coccygeal chordomas

These present with the rapid appearance of an eventually ulcerated subcutaneous mass [37–39] occasionally massive within the presacral space. Perineal pain, radicular pain [39, 40] or cauda equina syndrome may occur [39, 40]. Bladder and bowel dysfunction is common through compression and/or invasion of the nerves of the cauda equina or by direct compression of the urinary tract and colon by the presacral mass [37, 41].

### Vertebral column chordomas

The predominant symptoms depend on the orientation and development of the tumour. In the majority of cases, posterior enlargement of the tumour causes compression of the spinal cord or cauda equina [17, 26, 42–45]. Anterior progression can present with respiratory dysfunction and/or dysphagia in those tumours of the cervical and thoracic regions [46]. Pain is common and rigidity and/or spinal deformity have been similarly reported [17, 43, 47, 48].

### Metastatic dissemination

The incidence of chordoma metastases is quite variable, from 8.6 to 58 % in children [15, 17–19]. There appears to be a link between their incidence and local recurrence [19, 49]. Metastatic spread seems to be the prerogative of the under 5-year-olds [16, 23, 50], and essentially concerns tumours of the sacro-coccygeal region or of the spinal column. Metastatic dissemination primarily occurs via the blood circulation, but also via the cerebrospinal fluid, either via the subarachnoid spaces [50, 51] or through ventricular shunting [52]. There is also the risk of metastatic tumour deposition through the surgical route [53]. The principle site of metastases is the lungs [15–17, 34, 51, 54–57], followed by bone [50, 57], lymph nodes (cervical, inguinal, sub-clavicular) [15, 17, 57, 58], skin [50, 59], liver [19, 50, 59] and anecdotally the brain and spinal cord [59], meninges [50, 51], heart [59], pleura [15], kidney [59] and suprarenal glands [17, 23].

### Aetiology

#### Embryology

The term “notochorde” comes from the ancient greek *noton* (back) and *chorde* (cord), literally “dorsal cord”. It is a dorsal tubular structure occurring in embryos of all chordates [60]. In vertebrates, the notochord is replaced during development by the vertebral column and part of the cranial base. It has long been considered that the *nucleus pulposus* was the point of origin of chordomas. However, the origin of chordomas from the vertebral bodies, their main localisations at the extremities of the cranio-vertebral axis [13, 15, 23] and the absence of brachyury (a specific marker of chordomas) in the intervertebral discs [61] place into doubt the link between chordomas and the *nucleus pulposus*.

The fact that chordomas arise from notochordal cells is supported by different arguments. Apart from their identical location, there exists morphological and immunophenotypical similarities between the cells of chordomas and those of the notochord [62].

In fact, chordomas arise from notochordal remnants from an incomplete involution of the notochord and different from “normal” remnants potentially found in the *nuclei pulposi*.

### Molecular biology

Publications on this subject are infrequent and not specific to childhood chordomas. The majority of chordomas demonstrate hypodiploidy or pseudodiploidy with numerous structural rearrangements. Chromosomal deletions are more frequent than gains. The more frequent cytogenetic anomalies are monosomy 1 and a gain on chromosome 7 [63].

The brachyury growth factor (growth factor T) is a specific marker of chordomas, implicated in notochordal development. Its locus 6q27 is frequently amplified within chordoma cells and its inactivation can block growth of chordoma tumour cell lines (U-CH1) *in vitro* [64].

Numerous tyrosine kinase receptors are richly expressed in chordoma cells, such as EGFR (epithelial growth factor) [65], α and β PDGFR (platelet-derived growth factor), c-KIT [66, 67] and IGF-1 receptor [68]. Tumour suppressor genes CDKN2A, CDKN2B [17, 69] and FHIT [69] may also be implicated in the oncogenesis of chordomas.

Mutation of tumour suppressor genes TSC1 et TSC2 is seen in patients with chordomas associated with Tuberous Sclerosis. This association is specific to the paediatric population. Chordomas in these patients occur in very young children, are frequently sacral with a better long term prognosis [70]. The genes TSC1 and TSC2, being of the mTOR pathway, have raised the possibility that this pathway is implicated in intracellular signalling for oncogenesis in chordomas.

### Diagnostic methods

#### Computerised tomography (CT) [71]

**Fig. 1:**
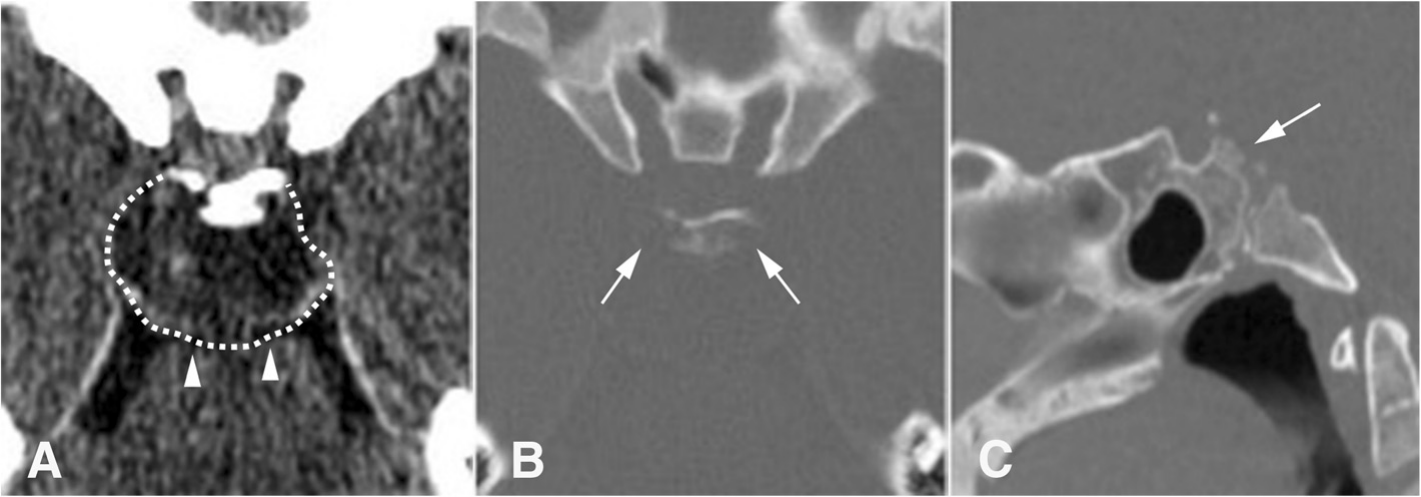
CT scanner of a clival chordoma. **a**. Axial brain window. The retroclival lesion developed posteriorly (dotted line) with contact to the brainstem (arrowhead). **b**. Axial bone window. Erosion of both posterior clinoid proccesses by the chordoma (arrows). **c**. Erosion of the posterior side f the clivus (arrow)

CT is essential for evaluating bony integrity and bone destruction of the skull base and also potential vertebral instability induced by tumour invasion (Fig. 1). In brain windows chordomas often appear heterogeneous due to the interspersing of tumour tissue (isodense), with areas of necrosis or myxoid cysts (hypodense). The hyperdensity frequently observed corresponds to bony sequestration from destruction of cortical and cancellous bone rather than tumoural calcification.

### Magnetic Resonance Imaging (MRI)

**Fig. 2:**
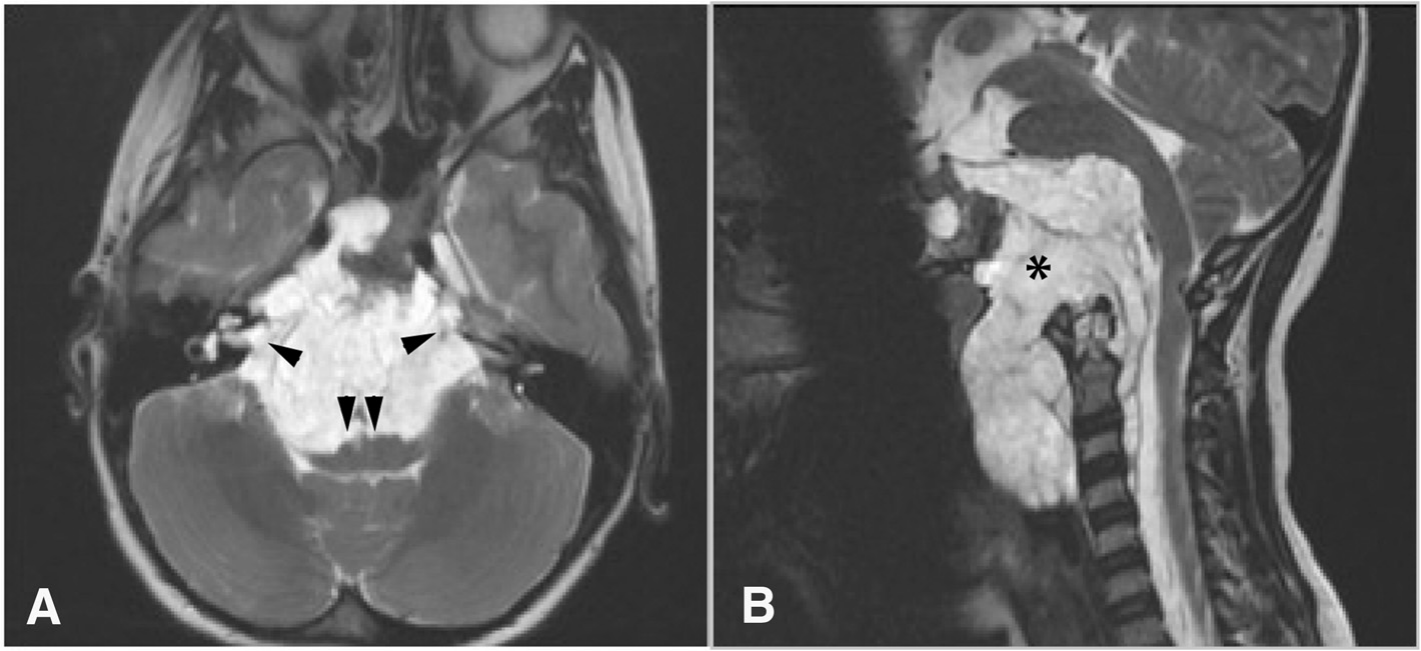
T2 weighted MRI of a clival chordoma. **a**. Axial plane. A voluminous chordoma developed from the clivus with extension to both cerebellopontine angles (single arrowheads). Brainstem is pushed back posteriorly (double arrowhead). **b**. Sagittal plane. The chordoma eroded the inferior part of the clivus (asterisk) and developed anteriorly to the cervical spine. Typical lobulated appearance with multiples hypointense septae can be observed

MRI provides accurate evaluation of the involvement of the adjacent soft tissues, including vasculature, cranial nerves, and changes in the brainstem related to tumour itself. Chordomas are iso/hypointense on T1-weighted images, with hyperintensity due to haemorrhage or cyst formation. The lesion is typically hyperintense on T2-weighted images, with a lobulated appearance and multiple hypointense septae (Fig. 2). The majority of intracranial chordomas demonstrate moderate to marked heterogeneous enhancement following contrast. If present, it is often heterogeneous, with a “honeycomb” appearance of the septae, whilst areas of necrosis or mucoid containing remain iso/hypointense.

### Angiography

Angiography is rarely useful but may provide additional informations to determine the relationship of the chordoma to vascular structures when equivoque on MR angiography. Exceptional temporary occlusion may be performed to determine the potential risk of vascular sacrifice during surgical procedures.

### Pathology

Macroscopically chordomas are blue or slate grey tumours, frequently soft and gelatinous. They are encapsulated nodular tumours that may contain areas of haemorrhage, necrosis or even cysts.

Chordomas have been divided into three histopathologic subtypes: typical or conventional chordomas, chondroid chordomas and dedifferentiated chordomas [1]. Microscopically, they have a characteristic architecture comprising of cells arranged in sheets or in lobules surrounded by an abundant myxoid extracellular matrix. Some tumour cells are quite large with numerous intracytoplasmic mucine or glycogen rich vacuoles: these are the characteristic physaliferous cells. Notably, approximately 10 % of chordomas do not contain physaliferous cells; the frequency of these cells can vary from 1 to 100 %. Generally, chordomas demonstrate a high degree of intratumoural heterogeneity [1]. Foci of intratumoural necrosis are frequent and often extensive. Mitoses, nuclear atypia and pleomorphisms are present to a variable degree.

Chondroid chordomas present an extracellular matrix that resembles those of hyaline cartilaginous tumours [1]. The distribution of the different histological entities can vary from simple chondroid foci dispersed within a predominantly chordoid structure to an equal distribution of the chondroid and chordoid components [13, 72].

Undifferentiated chordomas are mixed tumours presenting features of classic chordomas in association with foci of osteosarcoma or of high grade undifferentiated malignant spindle cell tumours [1].

Conventional and chondroid chordomas express epithelial markers including cytokeratins (notably CK 8, 18 and 19), Epithelial Membrane Antigen (EMA), S-100 protein, and specifically brachyury. On the contrary, undifferentiated chordomas lack reactivity for these epithelial markers. Yadav et al. observed that paediatric chordomas more frequently show p53 expression and loss of INI1, and have higher MIB-1 labelling index compared to their adult’s counterparts [73].

The distinction between these diverse chordoma tumour entities is not yet fully elucidated. Immunohistochemistry and molecular biological markers will probably contribute to classify these tumour variants.

### Differential diagnosis

- Benign notochordal cell tumour. These entities could be benign forms of chordoma [74]. Differential diagnosis is made on clinical and histological criterions [75].
- Chondrosarcomas. Tumours of the median axis, chondrosarcomas have more likely linear or globular calcifications and do not express epithelial markers nor brachyury [76].
- Chordoid meningiomas. A differential diagnosis of intradural chordomas.

### Genetic counselling

There is currently no particular genetic counseling as only few familial chordoma cases have been reported with different genetic abnormalities [77–86]. Variable losses of 1p [82, 83], 7q LOH [86] and a unique duplication of a region on 6q27 containing only the T gene (brachyury) [85] have been described.

### Management including treatment

Management of chordomas is multidisciplinary and because of the rarity of this disease, should ideally be performed in a specialized centre. Due to the high risk of relapse, management of this disease must be aggressive. The advent of MRI, the advances in neurosurgery (endoscopy) and the contribution of proton beam therapy have allowed considerable prolongation of life for these patients. Indeed, when comparing the survival rates of children treated before and after 1990 in the literature, there is a statistically significant difference in overall survival with the more recent cases having a better outcome (p = 0,001, log rank; data available for 153 cases from a review of 249 cases of intracranial and spinal chordomas). Today, treatment relies on as complete as possible surgical resection followed by adjuvant radiotherapy by proton therapy. Standard chemotherapy has no role even if certain authors have utilized chemotherapy with occasionally encouraging results.

### Surgery

Surgery is the first and essential step in the treatment of chordomas. The objectives are twofold: (1) maximal reduction in tumour volume with obtaining a macroscopically complete excision and (2) to remove any possible tumour residual away from neurovascular structures (spinal cord, brainstem, large vessels, internal auditory meatus, optic pathways and hypophysis) to maximize radiotherapy dose whilst minimizing secondary effects.

As in adults, all authors agree that the largest surgical resection possible must be achieved at the initial surgery [11, 13, 19, 87–90]. This attitude is commonly accepted, even if the paediatric series in the literature are too little to allow a real statistical analysis. Ridenour observed a better survival following a complete excision versus an incomplete excision in 35 children, without achieving statistical significance [18]. The localization of these tumours and the complexity of their extension allows a complete resection in the minority of cases [7, 91] due to the proximity to neural structures (cranial nerves, brainstem, sacral nerves) or vascular structures. The rate of complete surgical excision remains low in the major paediatric series published and varies between 0 % to 36.4 % [11, 17–19]. Maximal tumour resection often requires many surgical attempts [92], in utilizing different surgical routes in one or more procedures. The majority of routes to the cranial base currently used in adults may be applied to children with little modification and are well tolerated [93].

Orthopaedic management may be necessary in order to prevent or treat instability when spine is involved [27, 45, 46, 48, 94].

### Radiotherapy

Although radiotherapy is currently an integral part of the treatment of adult chordomas, issues regarding both the timing and optimal type of radiotherapy are largely unresolved in the literature [95]. Besides, very few data are available concerning the paediatric population and no comparative study has proven the benefit of radiotherapy following surgical resection in the outcome of paediatric chordomas.

The series published by Wold et al. is the only one that assessed the impact of conventional radiotherapy in paediatric chordomas [11]. Twelve patients (mean age 13.6 years) were treated for intracranial chordomas with a total or partial resection, followed in ten cases by conventional radiotherapy. After a mean follow-up of 67 months, two patients had died from disease, and the overall survival reached 75 % at 5 years. All patients alive at last follow-up had benefited from the association of surgery and radiotherapy. In a more general point of view, Borba et al., in a review of intracranial chordomas, confirmed that surgical excision, whether complete or incomplete, followed by radiotherapy (type not precise), offered a better outcome than surgical excision alone (p = 0.004, outcome of each group not precise) [16]. Actually, the majority of authors have recommended adjuvant radiotherapy [6, 42, 96, 97] after as complete as possible tumour resection.

The necessity of high dose radiotherapy in the treatment of chordomas [98], and the numerous potential complications of radiotherapy in growing children (pituitary deficiency, cognitive and neurosensory impairment, brain radionecrosis, necrosing leucoencephalopathy, fertility disorder, growing disorders) [99] lead to consider proton therapy as a radiotherapy modality of choice for paediatric chordomas, as it reduces by a factor of 2–3 the dose delivered to neighbouring structures [100]. Overall survival rates from 60 to 89 % after 5 to 7.25 years have been observed after treatment of cranial base and cervical chordomas by surgical removal followed by proton therapy (eventually associated with conventional radiotherapy) [19, 20, 101–103]. Side effects described in the different series published seem to be limited compared to conventional therapy. They are essentially represented by hypopituitarism, hypoacousy or worsening of anterior visual deficit; temporal lobe necrosis, cerebellar and brainstem parenchymal damage have also been punctually reported [19, 101–104]. Proton beam therapy may be used in young children, eventually under general anaesthesia [105]. It has been reported proton irradiation of skull base tumours in patients less than one year of age [106]. Although good outcomes have been reported in the series of patients treated with proton therapy, no study directly compared the results of this radiotherapy modality to those of conventional therapy.

### Chemotherapy

Similarly to adults, utilisation of chemotherapy in the management of paediatric chordomas is anecdotal with only 20 or so cases reported in the literature [11, 17, 19, 58, 59, 80, 107–109]. Some authors consider that chemotherapy used for sarcomas can also be used in undifferentiated chordomas [59, 110], such as ifosfamide and etoposide [58, 107] or doxorubicine [107, 109]. Whatever the agent used, chemotherapy has been used after relapse or in some metastatic cases with disappointing results. One publication reports a brief case of a 7-month old infant treated for a clival chordoma who had a durable complete response with chemotherapy alone (including vincristine, doxorubicin, cyclophosphamide, ifosfamide, etoposide and carboplatin) [111], but no data exist on the potential benefit of chemotherapy before any surgery.

Based on recent studies in molecular biology, the tendency in oncology has become to use targeted therapies as adjuvant treatment. Unfortunately, only few adult series have been available so far in the field of chordomas. Symptomatic and radiological improvement has been observed in adult treated by Gleevec® (Imatinib, tyrosine kinase inhibitor). This molecule has also been infrequently used in children without encouraging result [67, 112, 113]. Inhibitors of the mTOR pathway (sirolimus) and inhibitors of EGFR (cetuximab/gefitinib) have also been used in cases of resistant chordomas [112] or metastatic chordoma of the sacrum [114]. These observations need to be confirmed through large cohort studies with sufficient follow up and would require genomic analyses of paediatric chordomas, which are probably different compared to their adult’s counterparts.

### Prognosis

Globally survival is better in children than in adults except for the aggressive form of chordomas occurring in children under 5 years of age. The overall survival rate in the major paediatric series in the literature varies between 56.8 to 81 % [16, 18–20] (Table 1). These results observed in the paediatric population are generally better than those seen in adults where the survival rate varies from 23 to 66 % [115, 116].

**Table 1:** Outcome of paediatric chordomas observed in the major paediatric series of the literature.

Author	Number of cases	Treatment			Mean follow-up in months (min-max)	Progression free survival (%)	Overall survival (%)
		Surgery	Radiotherapy	Chemotherapy			
Benk et al.^[^ 19 ^]^	18	Partial resection (18)	Proton (18)	-	72 (19–120)	63 (5 years)	68 (5 years)
Borba et al.^[^ 16 ^]^	79 (review)	Not precise	Not precise	Not precise	39 (1–300)	-	56.8
Hoch et al.^[^ 20 ^]^	73	Total/Partial resection (73)	Proton (73)	-	90 (12–252)	-	81
Ridenour et al.^[^ 18 ^]^	20	Partial (14)	Conventional (10)	(2)	129 (1–501)	-	63
			Proton (2)				
			None (2)				
		Total (4)	Conventional (2)				
			None (2)				
		Biopsy (2)	Conventional (2)				
Necker - Lariboisière (not published)	34	Partial (24)	Conventional (2)	(3)	78 (0.3-239)	54.3 (15 years)	63
			Proton (3)				
			Conventional + Proton (12)				
			Radiosurgery (1)				
			Tomotherapy (1)				
			None (4)				
			Unknown (1)				
		Total (6)	Conventional (2) Proton (3) Conventional + Proton (1)	(1)			
		Unknown (4)					

Histological subtype is probably the main prognostic factor. Atypical and undifferentiated forms have clearly a worst outcome, compared to classic and chondroïd chordomas. Mortality rates for atypical (undifferentiated/poorly differentiated) forms vary from 67 to 83 %, compared to 14 to 27 % mortality rates for classic and chondroïd forms [18, 20].

The localisation of the tumour may also influence the prognostic. Intracranial lesions are considered to have a better outcome than those in the spinal column, which have a better outcome than those in the sacro-coccygeal region [18, 19, 117].

Finally, a major prognostic factor identified in chordomas was age of onset and it is notable that the worst progression occurs in very young children, under the age of 5 years [16, 17, 23, 50, 102, 118] ; the review of intracranial chordomas by Borba is quite clear on this subject [16]. Apart from a few cases, the majority of children less than 5 years reported in the literature died within 18 months following diagnosis despite surgery, radiotherapy and/or chemotherapy [15, 23–25, 50, 53, 58, 109, 117]. Age, where children under 5 years are more prone to develop more aggressive tumours, however, is not the only factor associated with a poorer outcome. It may be explained by the frequency of sacro-coccygeal localisations and atypical forms, and a more frequent metastatic dissemination.

### Unresolved questions

As for their adult’s counterparts, it is now largely admitted that surgery is the first step of the treatment of paediatric chordomas, and that as complete as possible removal has to be reached in order to improve the outcome. Interrogations still persist on the necessity of adjuvant therapy and its modality. Indeed, the usefulness of radiotherapy after total resection of paediatric chordoma is still not established and would deserve prospective analyses in international trials. One may ask if radiotherapy is compulsory after complete resection of a classical chordoma without invasion of functional structures. Moreover, data from the molecular biology will probably help us to distinguish in the future chordomas with a better prognosis that could be eligible to a surgical treatment alone. Although proton therapy has shown good outcomes in different studies, its superiority to other radiotherapy modalities has also to be proven. On the contrary, chemotherapy has shown disappointing results. Recent studies on biomolecular and genetic analyses of chordomas lead to the identification of promising targetable pathways. Use of such new targeted therapies has shown encouraging results in adults in case of advanced disease, but few paediatric patients have been included in these studies. Complementary studies will be necessary in order to assess their role in the treatment of paediatric chordoma, either as neoadjuvant treatment, or in case of progressive disease after standard treatments.

## Conclusions

Paediatric chordomas are rare malignant tumours with a high rate of recurrence. While prognosis is better than in adults, patients less than 5 years of age generally present with very aggressive disease and poor outcome. The surgical removal of the tumour still is the first step of the treatment and has to be as complete as possible. As for adults, proton beam therapy is the adjuvant treatment of reference, even if its benefit in some cases may be discussed. The role of chemotherapy has not been proven, but advances in genetics and molecular biology may help to develop targeted therapies during the next decades.

## Abbreviations

CT: computerised tomography
EGFR: epithelial growth factor
ICP: intracranial pressure
MRI: magnetic resonance imaging
PDGFR: platelet-derived growth factor
